# Detection of glucose-derived d- and l-lactate in cancer cells by the use of a chiral NMR shift reagent

**DOI:** 10.1186/s40170-021-00267-4

**Published:** 2021-11-06

**Authors:** Eul Hyun Suh, Carlos F. G. C. Geraldes, Sara Chirayil, Brandon Faubert, Raul Ayala, Ralph J. DeBerardinis, A. Dean Sherry

**Affiliations:** 1 Advanced Imaging Research Center, University of Texas Southwestern Medical Center, Dallas, TX USA; 2 Department of Life Sciences and Coimbra Chemistry Center, Faculty of Science and Technology, University of Coimbra, 3000-393 Coimbra, Portugal; 3 CIBIT - Coimbra Institute for Biomedical Imaging and Translational Research, University of Coimbra, 3000-548 Coimbra, Portugal; 4 Children’s Medical Center Research Institute, University of Texas Southwestern Medical Center, Dallas, TX USA; 5 School of Health Professions at Yvonne A. Ewell Townview Center, Dallas, TX USA; 6 Howard Hughes Medical Institute, University of Texas Southwestern Medical Center, Dallas, TX USA; 7 Department of Pediatrics and Eugene McDermott Center for Human Growth and Development, University of Texas Southwestern Medical Center, Dallas, TX USA; 8 Department of Chemistry and Biochemistry, University of Texas at Dallas, Richardson, TX USA; 9 Department of Radiology, University of Texas Southwestern Medical Center, Dallas, TX USA

**Keywords:** d- and l-lactate, Shift reagent-aided NMR, Cancer cells

## Abstract

**Background:**

Excessive lactate production, a hallmark of cancer, is largely formed by the reduction of pyruvate via lactate dehydrogenase (LDH) to l-lactate. Although d-lactate can also be produced from glucose via the methylglyoxal pathway in small amounts, less is known about the amount of d-lactate produced in cancer cells. Since the stereoisomers of lactate cannot be distinguished by conventional ^1^H NMR spectroscopy, a chiral NMR shift reagent was used to fully resolve the ^1^H NMR resonances of d- and l-lactate.

**Methods:**

The production of l-lactate from glucose and d-lactate from methylglyoxal was first demonstrated in freshly isolated red blood cells using the chiral NMR shift reagent, YbDO3A-trisamide. Then, two different cell lines with high *GLO1* expression (H1648 and H 1395) were selected from a panel of over 80 well-characterized human NSCLC cell lines, grown to confluence in standard tissue culture media, washed with phosphate-buffered saline, and exposed to glucose in a buffer for 4 h. After 4 h, a small volume of extracellular fluid was collected and mixed with YbDO3A-trisamide for analysis by ^1^H NMR spectroscopy.

**Results:**

A suspension of freshly isolated red blood cells exposed to 5mM glucose produced l-lactate as expected but very little d-lactate. To evaluate the utility of the chiral NMR shift reagent, methylglyoxal was then added to red cells along with glucose to stimulate the production of d-lactate via the glyoxalate pathway. In this case, both d-lactate and l-lactate were produced and their NMR chemical shifts assigned. NSCLC cell lines with different expression levels of *GLO1* produced both l- and d-lactate after incubation with glucose and glutamine alone. A *GLO1*-deleted parental cell line (3553T3) showed no production of d-lactate from glucose while re-expression of *GLO1* resulted in higher production of d-lactate.

**Conclusions:**

The shift-reagent-aided NMR technique demonstrates that d-lactate is produced from glucose in NSCLC cells via the methylglyoxal pathway. The biological role of d-lactate is uncertain but a convenient method for monitoring d-lactate production could provide new insights into the biological roles of d- versus l-lactate in cancer metabolism.

**Supplementary Information:**

The online version contains supplementary material available at 10.1186/s40170-021-00267-4.

## Background

Glycolysis is the major pathway for the conversion of glucose to pyruvate in all mammalian cells. Under aerobic conditions, pyruvate is transported into mitochondria and oxidized in the TCA cycle whereas in tissues with limited oxygen availability, pyruvate is converted to lactate in the cytosol and exported from cells. Lactate production even in the setting of adequate oxygen (the Warburg effect) is observed in cultured cancer cells and in tumors. Lactate can also be imported and used as a respiratory fuel in some tumors [1]. In terms of whole-body metabolism, Hui, et al. [2] have determined in fed and fasted mice that the circulatory turnover of lactate is even higher than glucose turnover. This suggests that lactate may be a more important substrate than glucose for whole-body metabolism. In the brain, it is widely accepted that glucose is the predominant source of energy, although glucose oxidation occurs indirectly, with astrocytes taking up glucose from the blood, converting it to lactate via glycolysis, then exporting lactate for oxidation by neurons [3, 4]. This means that lactate must be converted back to pyruvate by neuronal lactate dehydrogenase (LDH) before being completely oxidized in the TCA cycle. These examples illustrate the concept of a lactate shuttle where lactate can freely be exchanged among cells, tissues, and organs under aerobic conditions [5]. One possible advantage of the astrocyte-neuron lactate shuttle in the brain is that it transfers an “extra” reducing equivalent from astrocytes to the more energy-demanding neurons. The amount of ATP generated from these extra reducing equivalents in neurons depends upon whether the lactate is converted to pyruvate via cytosolic LDH or, as has been reported in some tissues, via mitochondrial LDH [6–8].

Given that both d-lactate and l-lactate can be produced from glucose in some mammalian tissues, one must consider the potential role of d-lactate in cell metabolism. Methylglyoxal (MG), a highly reactive three-carbon glycating metabolite formed at the level of the triose phosphates [9], is rapidly converted to S-lactoylglutathione by glyoxalase-1 (GLO-1) and subsequently to d-lactate by glyoxalase-2 (GLO-2) to eliminate this reactive species in glycolytic cells. The amount of d-lactate measured in the plasma, liver, and skeletal muscle of rats is quite variable (nM to μM) depending upon nutritional state and presence of diabetes [10]. The amount of d-lactate in liver tissue is consistently about 15–17% that of l-lactate [10]. More recently, de Bari et al. [11] demonstrated that d-lactate is transported into isolated rat liver mitochondria and converted to pyruvate using a membrane-bound FAD/FMN flavoprotein on the matrix side of the inner mitochondrial membrane. The resulting pyruvate formed from d-lactate can be oxidized or converted to oxaloacetate and transported out of mitochondria via a d-lactate/OAA antiporter. Thus, both d-lactate and l-lactate can serve as three-carbon precursors of gluconeogenesis.

**Scheme 1:**
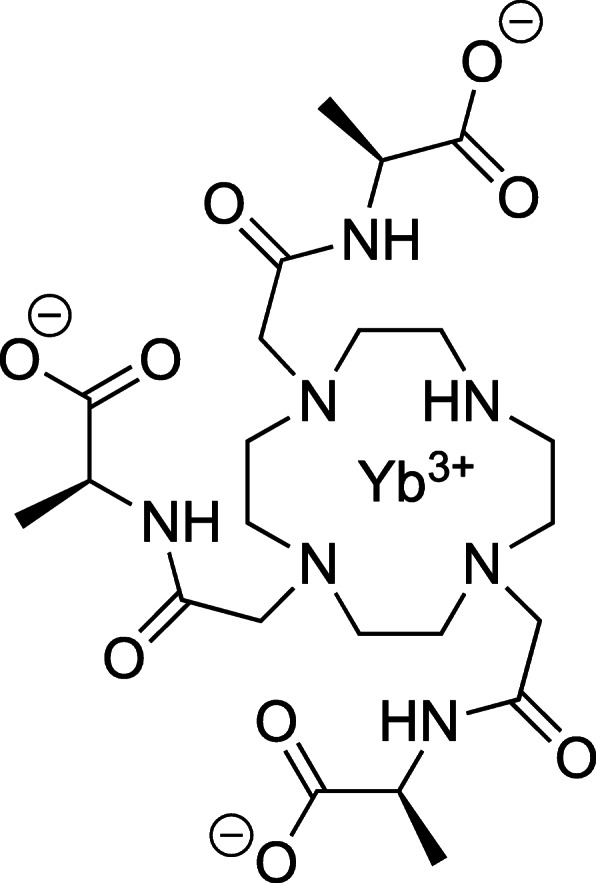
The chemical structure of the YbDO3A-(L-alanylamide) drawn as the (S)-isomer

Why is d-lactate often ignored in modern biochemistry textbooks and by scientists investigating the role of the Warburg effect in cancer? One likely reason is that d- and l-lactate are difficult to distinguish using modern spectroscopic methods such as NMR or MRI. Kuchel et al. [12] demonstrated that one can use stretched gelatins in NMR tubes to resolve the NMR methyl resonances of d- and l-lactate based upon the differential dipolar splitting seen in their CH_3_ resonances. Although the method is quantitative, it is not easily adapted to routine measures of d- and l-lactate in biological samples. Hyperpolarized ^13^C-labeled MG has also been used to detect d-lactate as an end-product of the glyoxalase pathway in RBCs, in EL4 tumor-bearing mice, and in the liver and brain of live animals [13]. This exciting observation demonstrates that the glyoxalase pathway is active in these tissues, but it does not allow measurement of the relative amounts of d-lactate versus l-lactate produced in those tissues without the addition of exogenous MG in supraphysiological quantities. We introduce here a simple NMR method for resolving the resonances of d- and l-lactate in any biological sample by the addition of a chiral shift reagent (SR). The water-soluble, chiral agent, YbDO3A-(L-alanylamide) (Scheme 1) (referred to as Yb_3_ in a prior publication [14]) forms a 1:1 complex with d- and l-lactate with nearly equal affinity and the chemical shifts of the CH and CH3 proton resonances in those two complexes are well-resolved (the methyl resonances of d- and l-lactate differ by ~9.5 ppm depending upon temperature). In this study, this SR-aided 1H NMR method was used to demonstrate production of d-lactate in RBCs exposed to methylglyoxal and production of both d- and l-lactate in two different human cancer cell lines exposed to glucose alone.

## Methods

### General

All reagents and solvents were purchased from commercial sources and used as received without further purification. Preparative HPLC was performed on a Waters Delta Prep system with a Waters Atlantis Prep, T3, OBD, 5 μm, 30 mm X 250 mm column pump coupled to a Waters diode array UV detector. LC-MS experiments were carried out on a Waters Alliance LC system with a Atlantis T3 column (C18, 5 μ, 250 × 4.6 mm), connected to a Waters diode array UV detector, and an electrospray ionization mass spectrometer (ESI MS) using a Waters Q TOF-MS-XEVO ESI positive mode for detection. ICP-OES analysis of Yb^3+^ (Galbraith Laboratories, Inc., Knoxville, TN) was used to calibrate the concentration of Yb_3_ in the final stock solutions.

### Preparation of shift reagent

Yb(III)-1,4,7,10-tetraazacyclododecane-1,4,7–tris(2-acetamido-L-alanine) (abbreviated Yb_3_) was prepared by mixing the ligand (prepared and purified as described previously[14]) with 0.95 equivalents of Yb(OH)_3_. The pH of the solution was adjusted to 5.5 and the reaction was stirred at room temperature overnight while the pH of the solution was maintained in the range of 5.5 – 6.0 by the addition of NaOH (or HCl). After filtration, the pH was adjusted to 7 with 1 N HCl and tested (xylenol orange) for free Yb^3+^ (none present). After filtering through a 2 μm membrane, the filtrate was lyophilized to give 0.60 g of Yb_3_ as a cream-colored solid. m/z (ESI-MS^+^): 731.02 (M+H)^+^ calculated for C_23_H_39_N_7_O_9_Yb 731.22.

### NMR spectroscopy

^1^H, ^13^C, and CEST spectra were recorded on 9.4T Varian VNMRS direct-drive console spectrometer operating at 400 MHz (^1^H) and 100 MHz (^13^C), respectively. ^1^H NMR spectra were collected using a 90° pulse, 10,000 Hz sweep width, 19,979 data points, 2 s acquisition time, and a 1 s interpulse delay at 25°C averaged over 512 scans. Spectra were analyzed using ACD/SpecManager (Advanced Chemistry Development, Inc., Toronto, Canada). CEST spectra of Yb_3_ were collected by measuring the bulk water proton intensity after a series of 5 s frequency-selective pulses were applied prior to collection of the water signal (8 s delay between scans). The saturation frequency was arrayed in steps of 400 Hz. CEST Z-spectra are presented as a percent reduction of the intensity of the water signal (M_z_/M_0_) versus saturation frequency.

### Red blood cell experiments

The fresh blood, collected from a donor under an approved university protocol, was centrifuged at 2000 rpm for 10 min at 4°C. The plasma, including the buffy coat layer, was removed. The packed erythrocytes were washed in phosphate buffer (10 mM phosphate, 137 mM NaCl, 2.7 mM KCl, pH 7.4), resuspended in phosphate buffer (40% hematocrit), and exposed to either 5 mM glucose, 5 mM MG, or a mixture of 5 mM glucose plus 5 mM MG. After incubation for 15 min, 1 h, or 2 h, a 0.5-mL of buffer was collected and centrifuged, and Yb_3_ was added to a supernatant to a final concentration of 2.2 mM. For detection of d- and l-lactate directly in a suspension of red blood cells, RBCs were resuspended in phosphate buffer (10 mM phosphate, 137 mM NaCl, 2.7 mM KCl, pH 7.4) and incubated with 5 mM glucose, 5 mM MG, or no added substrate. After an incubation period of 2h, Yb_3_ was added to the 0.5 mL of packed RBCs at a final concentration of 2 mM.

### Lung cancer cell lines

H1395 and H1648 cells were cultured in 100-mm plates in RPMI-1640 medium (Sigma, R8758) supplemented with 10% of dialyzed fetal bovine serum (FBS) (Sigma, F2442) and 20 units of penicillin-streptomycin (Sigma, P0781). The cells were starved for 12 h prior to the initiation of the experiment, then washed three times in phosphate-buffered saline (PBS) and incubated in a basal medium (Sigma, D5030) supplemented with 5 mM glucose or 5 mM MG for 4 h. The culture medium (10 mL) was then lyophilized and redissolved in 0.4 mL of buffer containing 5 mM Yb_3_ for NMR analysis. 3553T3 parental cells, a *Glo1*-deleted clone, and cells in which *Glo1* was re-expressed were cultured in 100 mm culture plates with DMEM medium (Sigma, D5796) supplemented with 10% of FBS and 20 units of penicillin-streptomycin. When the cells reached confluence, the cells were washed with phosphate buffer and incubated for 4 h in basal medium (Sigma, D5030) supplemented with 5 mM MG and 5 mM glucose. As before, the culture media (7 mL) was then lyophilized and redissolved in 0.4 mL of buffer containing 10 mM Yb_3_ for NMR analysis. The number of cells used in each experiment (~3 x 10^6^ for H1395 and H1648 cells and ~1.5 x 10^6^ for 3553T3-derived cells) was determined using a TC20 Automated Cell Counter (Bio-Rad).

### Western blots

Cells were lysed in RIPA buffer supplemented with the following additives: protease and phosphatase tablets (Roche), DTT (1 μg/ml), and benzamidine (1 μg/ml). Cleared lysates were resolved by 4–20% SDS-PAGE gels, transferred to nitrocellulose, and incubated with primary antibodies against Glyoxalase I (Novus Biologicals, NBP1-19015) and Beta Actin (Cell Signaling, 4970). Immunoreactive proteins were visualized by chemiluminescence (Pierce, 32106).

## Results

### Quantitative analysis of d- and l-lactate by NMR

**Fig. 1:**
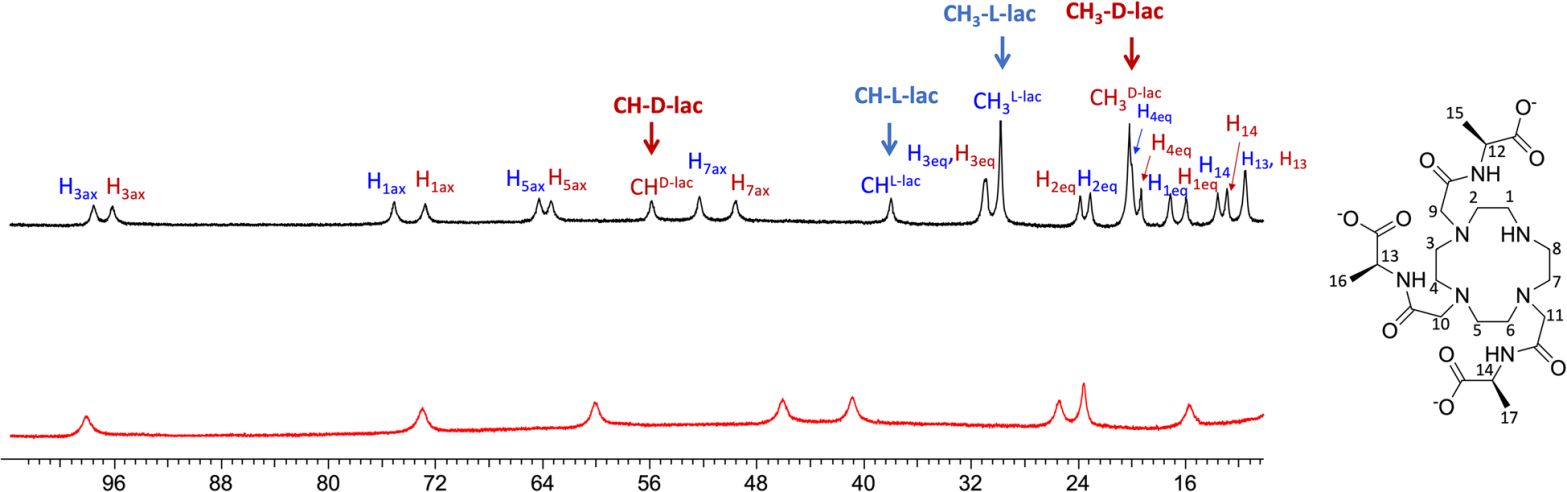
^1^H NMR spectra of an aqueous solution of Yb_3_ (6.3 mM) (bottom) and upon addition of d-lactate (15 mM) and l-lactate (15 mM) to the Yb3 sample (top), 9.4T; pH 7.2; 298K. The ^1^H NMR chemical shifts of all l-lactate ▪ Yb_3_(blue) and d-lactate▪ Yb_3_(red) protons were assigned as shown by the colored labels. The protons of the excess non-chelated lactate are far upfield in their normal diamagnetic positions (not shown here)

The fundamental basis of enantiomeric detection and quantification of d- and l-lactate by NMR was to add YbDO3A-(L-alanylamide)_3_ (abbreviated Yb_3_) as a paramagnetic shift reagent to biological samples [14]. Yb_3_ was selected over other amino acid amide options for several reasons: (1) ease of synthesis; (2) Yb_3_ exists in solution as a single stereoisomer (Λ(δδδδ) or Δ(λλλλ)) which, upon formation of a complex with lactate, yields single sharp CH and CH_3_ resonances for both d- and l-lactate; and (3) the paramagnetic properties of Yb_3_ result in large chemical shift differences between the bound resonances of d- and l-lactate [14]. The high-resolution ^1^H NMR spectrum of Yb_3_ used in this study is shown in Fig. 1. The downfield proton resonances in this complex, previously assigned using 2D EXSY NMR [14], reflect individual ligand protons in this highly asymmetric complex. Upon addition of either d- or l-lactate, two new proton resonances appear in the spectrum reflecting the methine and methyl protons of d-lactate·Yb_3_ or l-lactate·Yb_3_. The methyl resonance of d-lactate·Yb_3_ does overlap somewhat with the H_4eq_ resonance of l-lactate·Yb_3_, so this needs to be taken into account in all quantitative measures. Fortunately, all of the highly shifted ethylenediamine protons split into two peaks, one reflecting d-lactate·Yb_3_ and the other reflecting l-lactate·Yb_3_, so the areas of these separated resonances provide an second analytical measure of the relative amounts of d- versus l-lactate in solution.

The upper panel of Fig. 1 shows the ^1^H spectrum of Yb_3_ after the addition of both d- and l-lactate (both in excess). The protons of unbound, excess lactate appear in their normal diamagnetic positions (Figure S2). This shows that the rate of lactate exchange between the lactate·Yb_3_ complexes and free lactate is slow in comparison to their frequency differences. The well-separated methyl protons (21.0 ppm and 30.4 ppm) and methine protons (56.7 ppm and 38.6 ppm) of d- and l-lactate, respectively, indicate that either pair of resonances or both pairs could be used to quantify the amount of d- versus l-lactate in biological samples. The near-equal intensities of the two methine or methyl resonances in the top spectrum of Fig. 1 suggest that the two enantiomers of lactate must have nearly equal binding affinities with Yb_3_.

To verify this, an additional ^1^H NMR titration experiment was performed. Upon the addition of increasing amounts of either d- or l-lactate (from 0 to 7 mM) to a fixed concentration of Yb_3_ aqueous solution (2 mM), the ^1^H-NMR signals of the bound lactate-methyl resonances gradually increased in intensity until a 1:1 complex was fully formed. At that point, further addition of lactate did not alter the intensity of the bound lactate methyl resonances further. These binding curves were fit to a simple 1:1 binding model (Equation S1 and Figure S1) [15] to yield dissociation constants (K_D_) of 914 ± 15 μM for d-lactate and 709 ±35 μM for l-lactate. Although the fitting results showed these binding constants did differ slightly as one would expect for molecules of differing chirality, this small difference translates to a correction factor of only 2–3% in the methine or methyl proton intensities of d- versus l-lactate in Fig. 1.

### Production of d- and l-lactate in erythrocytes

**Fig. 2:**
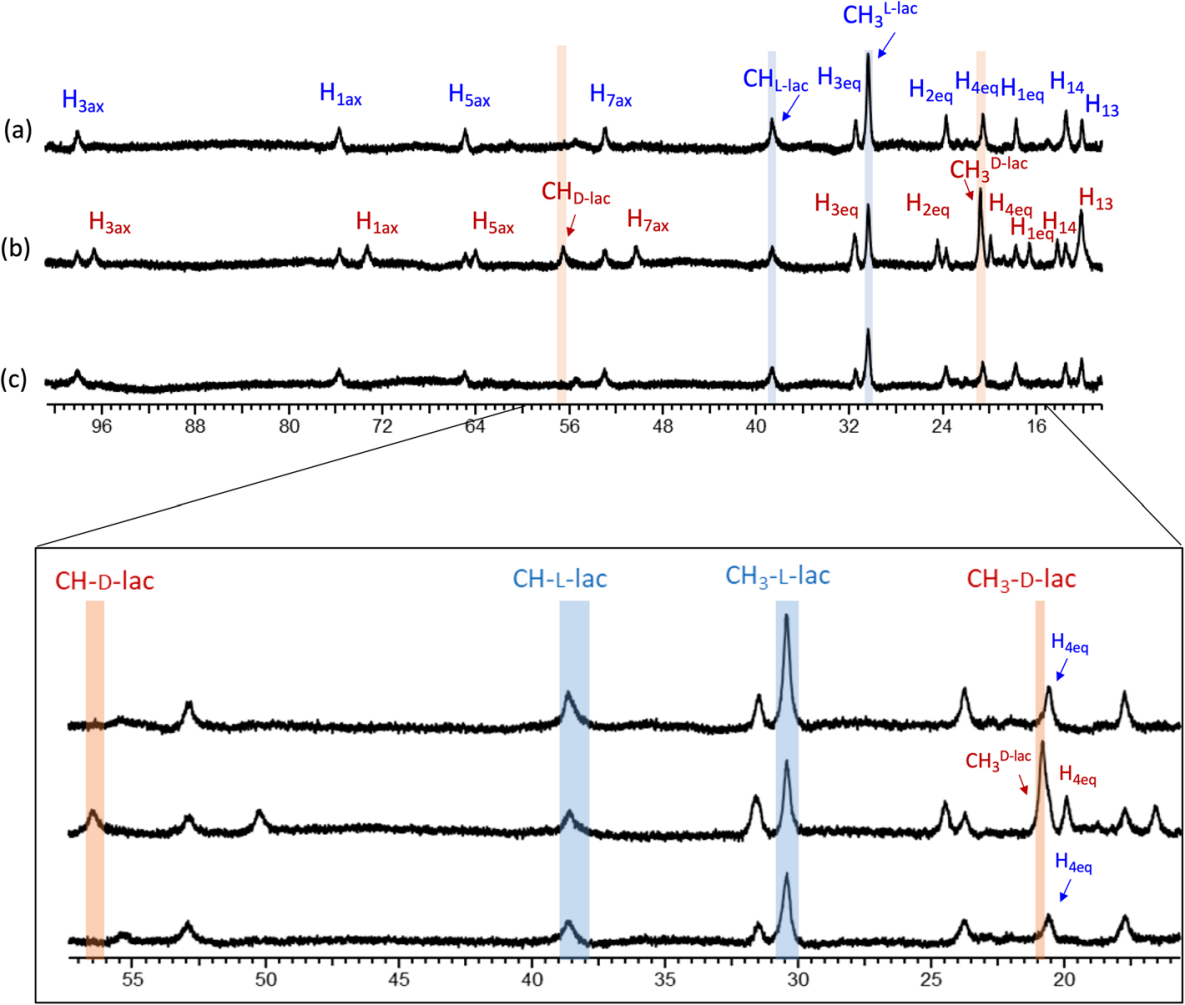
Detection of d- and l-lactate formation in erythrocytes using Yb_3_ (2 mM) by ^1^H NMR e-buffered saline, pH 7.4 after incubation for 2 h at 37°C with **a** 5 mM glucose, **b** 5 mM MG, and **c** wo/substrate. The highlighted methyl and methine resonances are assigned to l-(blue) and d-(red) lactate

Erythrocytes are known to have a glyoxalase pathway that converts methylglyoxal to d-lactate via glyoxalase 1 (GLO1) and glyoxalase 2 (GLO2) using glutathione as a co-factor [16]. To examine whether Yb_3_ detects both l-lactate and d-lactate production in human RBCs suspension, freshly isolated RBC suspensions in phosphate buffer (40% hematocrit) were incubated with either (a) 5 mM glucose, (b) 5 mM MG, or (C) no substrates. After incubation for 2 h at 37°C, a 2 mM Yb_3_ was added to the RBCs suspension before collection of their NMR spectra (Fig. 2). The spectrum in Fig. 2 a shows that 1.3 mM l-lactate was produced from glucose in RBCs; no d-lactate was evident in this spectrum. However, in RBCs incubated with MG alone, a significant amount of d-lactate (1.2 mM) was produced along with about 0.8 mM l-lactate (Fig. 2 b). Here, l-lactate must have been produced from intermediates remaining in the washed RBCs. In the RBC sample incubated without added substrates, nearly the same amount of l-lactate (1.1mM) was again produced from glycolytic intermediates (Fig. 2 c). Similar results were found in separate experiments where RBCs were incubated with these same substrates and the supernatants were separated from cells prior to the addition of the SR (Figure S3 and S4). This suggests that Yb_3_ remains extracellular in the experiments with cells present. We also collected CEST spectra of the supernatant samples and the areas of the CEST peaks for d- versus l-lactate⋅Yb_3_ complexes [14] gave quantitative values for D-Lac/L-Lac very similar to those measured by ^1^H NMR (Figure S5). The disadvantage of CEST spectroscopy for this measurement in comparison to ^1^H spectroscopy is that absolute concentrations are more difficult to obtain because the CEST intensities not only depend upon concentration but also the intensity of the applied B_1_ field and the amount of time the applied B_1_ is applied.

### Production of d- and l-lactate in lung cancer cells

**Fig. 3:**
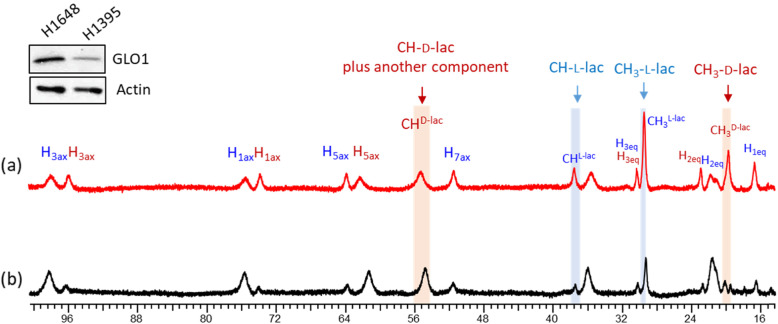
1^1^H NMR spectra of supernatant collected from **a** H1648 cells and **b** H1395 cells after incubation with glucose and glutamine for 4 h. After 4 h, 10 mL of media was collected, lyophilized, and redissolved into 0.4 mL water containing 5 mM Yb_3_ for analysis by ^1^H NMR (FA=45°, 9.4 T). The highlighted methyl and methine resonances are assigned to l-(blue) and d-(red) lactate. The Western blots (inset) show differences in *GLO1* expression in H1648 and H1395 cells

*GLO1* has been reported to be overexpressed in several human cancer cell lines including leukemia, melanoma, prostate cancer, and breast cancer cells [17–20] and has been implicated in cancer progression and drug resistance [21, 22]. A recent study showed that expression of *GLO1* is required for the growth of human-derived NSCLC xenografts in mice [23]. *GLO2* also plays a role in androgen-dependent tumorigenesis in prostate cancer regulated by p53 [24]. To examine whether this SR-aided NMR-based method can be used to monitor the production of d- and l-lactate in tumor cells, two different cell lines with high *GLO1* expression (H1648 and H 1395) were selected from a panel of over 80 well-characterized human NSCLC cell lines [25]. These cells were cultured in 5 mM glucose and glutamine added to RPMI media, grown to confluence, then washed and incubated with a modified buffer containing only 5 mM glucose and glutamine for sustained growth. After an additional 4 h, the supernatant was collected, freeze-dried, and dissolved in 0.4 mL of water containing 5 mM Yb_3_. The resulting ^1^H NMR spectra (Fig. 3) show that both cell lines make appreciable d-lactate in an apparent *GLO1* expression-dependent manner (d-lac/L-lac = 0.63 in H1648 cells and 0.26 in H1395 cells).

### d-lactate production in GLO1-deleted and re-expressed clones

**Fig. 4:**
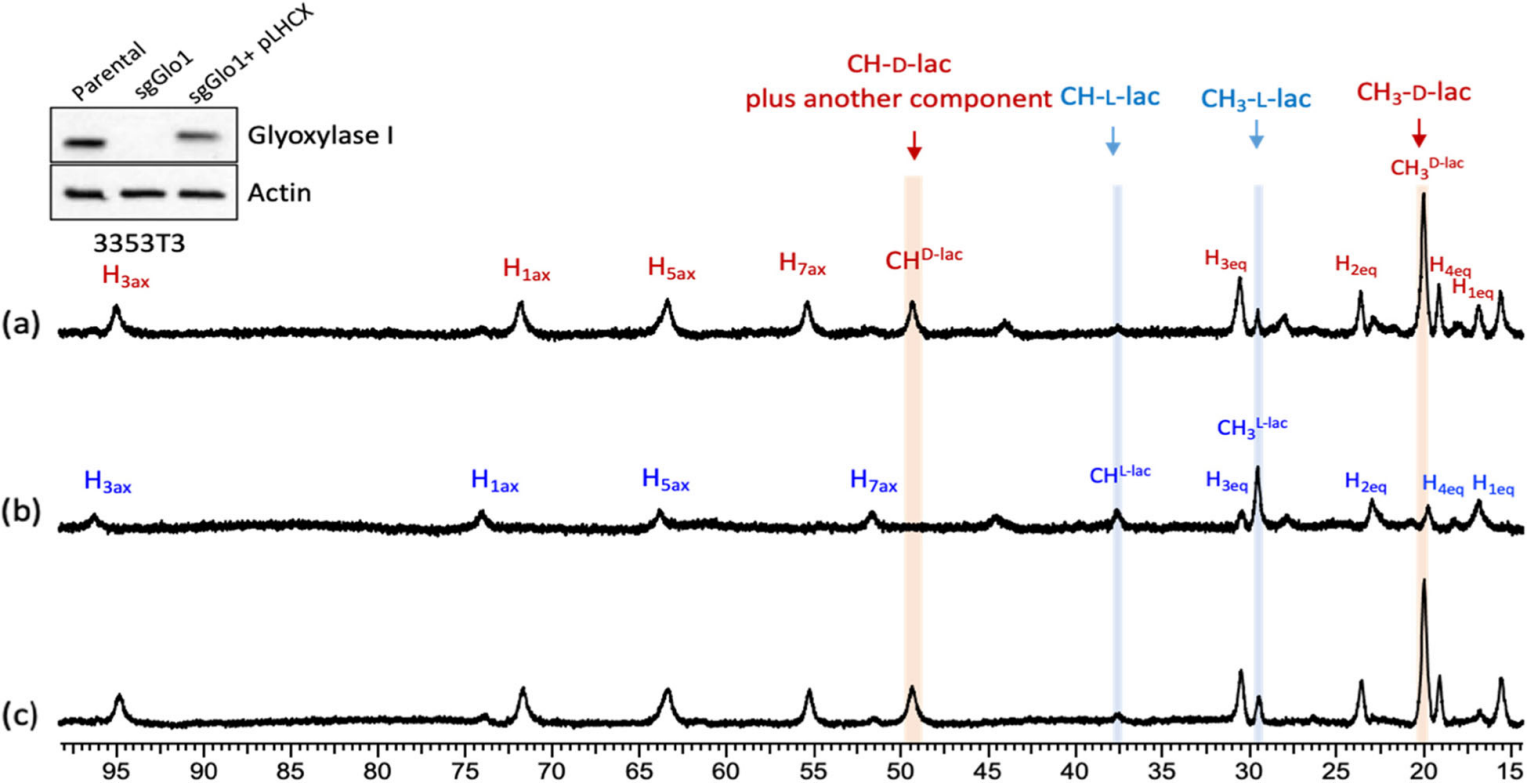
^1^H NMR (400MHz) spectra of media collected from **a** sg*Glo1* pLHCX cells (re-expressed GLO1), **b** sg*Glo1* (GLO1 deleted cells), and **c** parental 3353T3 cells. Each cell line was incubated with 5 mM methylglyoxal (MG) in DMEM basal media for 4h, 37°C, and pH 7 and mixed with 10-mM Yb_3_ prior to collection of the NMR spectra. The highlighted methyl and methine resonances are assigned to l-(blue) and d-(red) lactate. Western blots showing GLO1 expression in 3353T3 cells

**Fig. 5:**
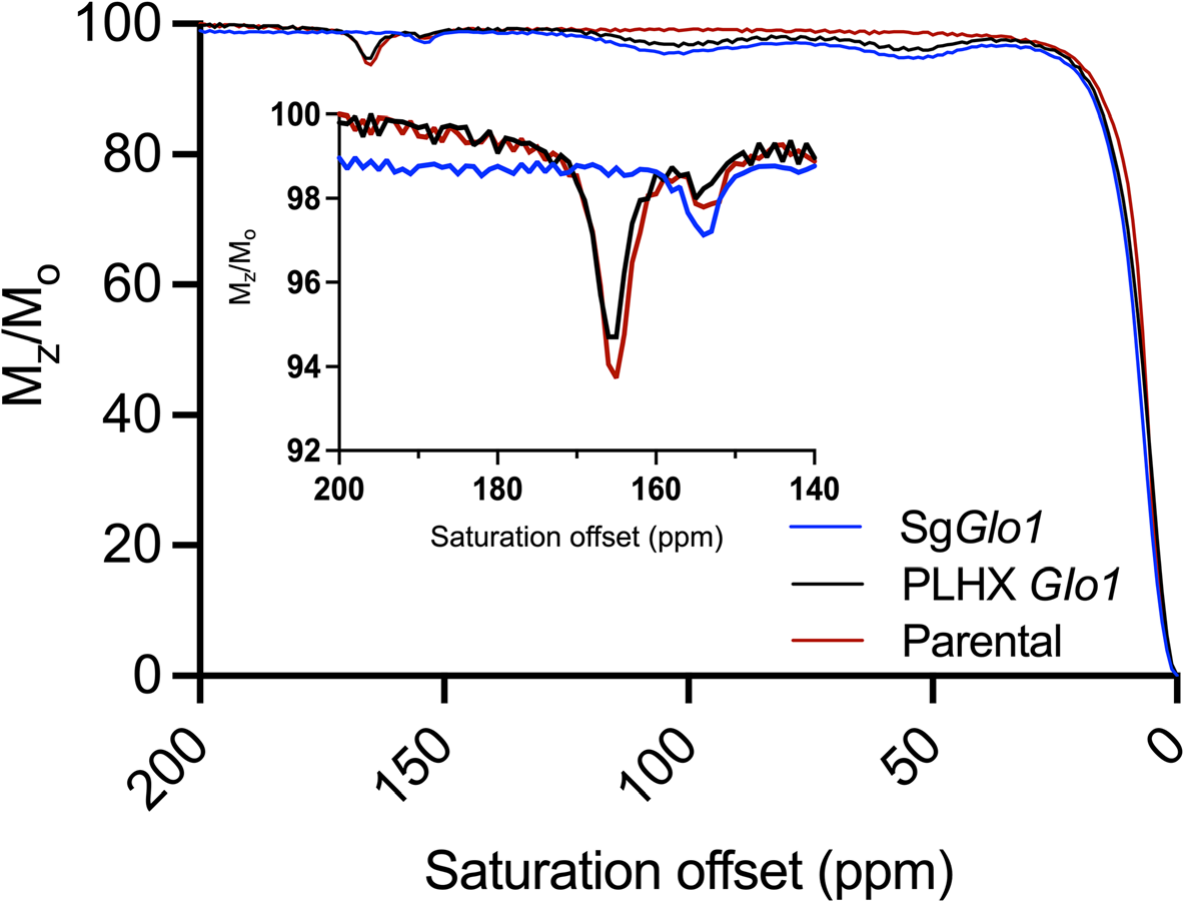
CEST spectra of the Yb_3_ complex (10 mM) containing 3353T3 cancer cell-cultured media which were obtained via incubation cells with 5 mM methylglyoxal (MG) in DMEM basal media (4 h at 37°C, pH ~ 7.0). The CEST peak at 165 ppm reflects d-lactate while the peak at 154 ppm reflects l-lactate in each cell line. Glo1 re-expressed cells, *sgGlo1* pLHCX (black), Glo1 deleted cells, *sgGlo1* (blue), and parental cells (red)

Additional experiments were designed to further test whether d-lactate production depends upon the expression of *GLO1*. Murine lung cancer cells (3553T3) expressing a guide RNA targeting *Glo1* (sg*Glo1*) to knock out expression of this gene, and an isogenic line in which *Glo1* was re-expressed (355T3 sg*Glo1* pLHCX *Glo1* )[23] were incubated in the presence of 5 mM MG for 4h. After this incubation period, 7 mL of supernatant was collected, freeze-dried, and redissolved in 0.4 mL of water containing 10 mM Yb_3_ for analysis by ^1^H NMR. As shown in Fig. 4, d-lactate was clearly evident in a *Glo1* expression level-dependent manner with high levels of d-lactate produced from MG in the parental 3553T3 and 355T3 sg*Glo1* pLHCX *Glo1* cells, and no d-lactate detection in sg*Glo1* cells. d-lactate was not detected after incubation with 5 mM glucose (Figure S6). The CEST spectra of these same samples (Fig. 5) reported identical d- and l-lactate ratios as those measured by ^1^H NMR.

## Discussion

Cancer promotes reprogramming of cell metabolism to support proliferation [26]. The highly glycolytic phenotype characteristic of most cancer cells has become an appealing therapeutic target [27, 28]. Elevated glucose metabolism inevitably produces more MG as a by-product [29, 30]. To counteract the toxic effects of MG, the glyoxalase pathway converts MG to d-lactate by sequential enzymatic reactions catalyzed by GLO1 and GLO2. Even though it has been reported that mammalian tissues are deficient in d-lactate dehydrogenase [31], Tubbs et al. showed that d-lactate is metabolized to pyruvate by the enzyme d-α-hydroxy acid dehydrogenase at a one-fifth rate of l-lactate [32]. A more recent study showed that prostate cancer cells metabolize d-lactate using d-lactate dehydrogenase, a mitochondrial membrane flavoprotein, the activity and protein level of which are higher in prostate cancer cells (PC-3) than in non-cancerous PNT1A cells [33]. Thus, detection of d-lactate in cancer in vivo could be important in that it represents a novel target for cancer diagnosis and prognosis of anti-cancer therapeutic strategies.

Potential applications of d-lactate detection are not limited to cancer metabolism. Although d-lactate is considered non-physiological in mammalian tissues, it does play a role in brain metabolism. The activity of the glyoxalase pathway is higher in astrocytes compared to neurons, consistent with higher glucose utilization and hence the greater requirement of cellular detoxification of MG in this cell type [34]. The link between MG cytotoxicity and advanced glycation end products (AGE) tightly correlates with the pathogenesis of the neurodegenerative disease [35]. A recent study showed that elevated levels of MG are present in the cerebrospinal fluid of patients with Alzheimer’s disease (AD) [36]. The *GLO1* level in AD was found to correlate with the progression of the disease, increasing in early stages and then decreasing in middle to late stages of AD [37]. Other pathophysiological conditions also associated with increased serum and urinary d-lactate production are diabetic ketoacidosis and small bowel resection. Furthermore, significantly elevated serum d-lactate was also reported in infection, ischemia, and trauma [38]. In particular, d-lactate is also elevated in various pathogenic bacteria that can be a marker of infections [39]. Thus, a diagnostic method for the detection of d-lactate could provide new insights into the metabolic role of d-lactate in various disease processes. Current methods used to detect d-lactate rely upon either an enzymatic assay or liquid chromatography tandem mass spectrometry coupled with a chiral column, methods limited to analytical detection of d-lactate in tissue or cell extracts [38, 40].

In this study, we demonstrated the feasibility of using Yb_3_ for enantiomeric discrimination of d- and l-lactate produced in erythrocytes and cancer cells. Both d- and l-lactate bind to open coordination positions on Yb_3_ in a bidentate fashion and with similar binding affinities. Although not measured, the rate of exchange of lactate on and off the complex is slow on the NMR timescale (Δω >> k_exch_), where Δω is the difference in chemical shift of the bound versus free resonances measured in Hz and k_exch_ is the rate of lactate exchange on and off the Yb_3_ complex. This results in distinct ^1^H NMR signals for unbound lactate versus Yb_3_-bound lactate. In the experiments described here, excess Yb_3_ was present in each NMR sample, so the relative intensities of the NMR signals of d- versus l-lactate directly reflect the quantitative levels of each enantiomer produced by cells during the incubation period. One of the nice features of this SR-aided technique is that one can quantify d- and l-lactate by integrating the areas of their well-separated methine or methyl resonances while taking advantage of the fact the ethylene signals of Yb_3_ also provide a second direct readout of the relative amounts of d-lactate:Yb_3_, l-lactate:Yb_3_, and unbound Yb_3_ present in each sample. A second nice feature is that the chemical shifts of the bound forms of lactate are independent of pH between 6 and 7.4 (Figure S7) so the method is applicable in cells that become acidic during the production of excess lactate.

Which other variables must be considered to ensure this method would provide quantitative measures of d- and l-lactate production in rodent tumor models? If one assumes that sufficient Yb_3_ can be injected into an animal to achieve an extracellular concentration of 1 mM, similar to that after a typical injected dose of a Gd-based contrast agent, and if the extracellular concentration of d- and l-lactate produced by a tumor at steady-state is 100 and 600 μM, respectively, then the ^1^H NMR signals would provide a direct readout of the absolute concentrations of d- and l-lactate. If, however, the local extracellular concentrations of d- and l-lactate are much higher, for example, 1 and 6 mM, then there would not be sufficient Yb_3_ present to fully form complexes with all lactate. However, even in this circumstance, the relative intensities of the methyl resonances of the d- and l-lactate complexes with Yb_3_ would reflect the relative amounts of the two enantiomers (after an appropriate correction factor) since the binding constants of the two complexes are nearly equal. Ultimately, the lower detection limits for detection of d- and l-lactate will be determined by the sensitivity of the imaging coil and field strength of the scanner used for ^1^H detection. The biocompatibility and toxicity of Yb_3_ are yet to be evaluated, but other lanthanide DOTA-amide-type complexes have proven safe for injection into animals at relatively high doses [41].

## Conclusions

The chiral shift reagent method presented here allows for rapid, simultaneous monitoring of d- and l-lactate production in cancer cells by either high-resolution ^1^H NMR or CEST NMR without additional chiral separation methods. Both isomers of lactate form bidentate complexes with Yb_3_ with nearly equal affinities and the paramagnetic SR properties of Yb_3_ reagent shift the proton resonances of d- and l-lactate well-downfield and well-separated from each other. Integration of the respective methyl resonances or the -OH CEST signals provides a quantitative measure of d- versus l-lactate. The method was used to measure the production of d- and l-lactate in two different human NSCLC cell lines over a 4-h period where it was shown that both cell lines produce an appreciable d-lactate from glucose in an apparent *GLO1* expression dependent manner. The biological significance of d-lactate production in these cells will require further studies, but the importance of the SR-aided NMR method reported here is that it offers other investigators a simple method for monitoring d- versus l-lactate production in living cells.

## Supplementary Information

**Additional file 1: Figure S1**. Titration curves for d- and l-lactate. **Figure S2**. Full ^1^H NMR spectrum of d-lactate (15 mM) and l-lactate (15 mM) addition to Yb_3_ (6.3 mM) showing the presence of excess free lactate CH_3_ and CH resonances in their normal diamagnetic positions (inserted). **Figure S3**. Detection of d- and l-lactate formation in erythrocytes using Yb_3_ (2.2 mM) by ^1^H NMR spectra of the supernatant of erythrocytes (40 % hematocrit) in phosphate buffered saline, pH 7.4 after incubation with (a) 5 mM glucose for 2 h (b) 5 mM methyl glyoxal for 2 h (c) 5 mM glucose for 30 min followed by addition of 5 mM MG for additional 1.5 h and (d) 5 mM methyl glyoxal for 2 h (d) wo/substrate for 2 h at 37°C. **Figure S4**. Detection of d- and l-lactate formation in erythrocytes at each time point during 2 h incubation using Yb_3_ (2.2 mM) by ^1^H NMR spectra of the supernatant of erythrocytes (40 % hematocrit) in phosphate buffered saline, pH 7.4 (a) 5 mM glucose for 2 h (b) 5 mM glucose for 30 min followed by addition of 5 mM MG for additional 1.5 h and (C) 5 mM methyl glyoxal for 2 h (d) wo/substrate for 2 h at 37°C. The data was normalized with no substrate incubation data as background correction. **Figure S5**. CEST spectra of the Yb_3_ complex (5mM) in RBC cultured media. Presaturation pulse of 5s with B_1_ of 15uT was applied at 298K using 9.4 NMR spectrometer: RBCs (40% hematocrit) was incubated in Phosphate-buffered saline (pH ~7.0, 2 h at 37 °C) with 5mM glucose; Glc 5mM, 5mM glucose 30min incubated then add 5mM methyl glyoxal; Glc + MG, and 5mM methyl glyoxal; MG 5mM. The CEST peaks are assignable at 168ppm to d-lactate and at 157ppm are l- lactate. **Figure S6**. ^1^H NMR (400MHz) spectra of media collected from (a) sg*Glo1* pLHCX cells (re-expressed GLO1) (b) sg*Glo1* (GLO1 deleted cells) and (c) parental 3353T3 cells. Each cell line was incubated with 5mM glucose in DMEM basal media for 4 h, 37 °C, pH 7. Subsequently, a 0.5 mL volume of media was collected and mixed with 10 mM Yb_3_ prior to collection of the NMR spectra. The highlighted methyl resonances are assigned to l-(blue) and d-(red) lactate. **Figure S7**. 1H NMR spectra of an aqueous solution of Yb_3_ (6 mM) in the presence of d-lactate (15 mM) and l-lactate (15 mM) at pH 6.0 and 7.4. The chemical shifts of CH_3_-L-lac and CH_3_-D-lac were insensitive to pH over this range. The signals of excess unbound d- and l-lactate appeared in their normal diamagnetic positions (not shown here). **Table S1**. ^1^H NMR shift (ppm) of l-lactate·Yb_3_ and d-lactate·Yb_3_ of RBCs supernatant sample.

## Data Availability

All data and materials are fully described in the manuscript. A copy of all data analyzed is available from the corresponding author upon reasonable request.

## Abbreviations

LDH: Lactate dehydrogenase
GLO-1: Glyoxalase-1
GLO-2: Glyoxalase-2
NMR: Nuclear magnetic resonance
SR: Shift reagent
EXSY: Exchange spectroscopy
CEST: Chemical exchange saturation transfer
MG: Methylglyoxal
Yb3: Yb(III)-1,4,7,10-tetraazacyclododecane-1,4,7–tris(2-acetamido-l-alanine)
AGE: Advanced glycation end products
