# Supplementary material for: Detection of glucose-derived d- and l-lactate in cancer cells by the use of a chiral NMR shift reagent
Source: Cancer Metab. 2021 Nov 6;9:38. doi: 10.1186/s40170-021-00267-4 (PMC8571830; doi:10.1186/s40170-021-00267-4)
Supplement: Supplementary file 1 — Additional file 1: Figure S1. Titration curves for d- and l-lactate. Figure S2. Full 1H NMR spectrum of d-lactate (15 mM) and l-lactate (15 mM) addition to Yb3 (6.3 mM) showing the presence of excess free lactate CH3 and CH resonances in their normal diamagnetic positions (inserted). Figure S3. Detection of d- and l-lactate formation in erythrocytes using Yb3 (2.2 mM) by 1H NMR spectra of the supernatant of erythrocytes (40 % hematocrit) in phosphate buffered saline, pH 7.4 after incubation with (a) 5 mM glucose for 2 h (b) 5 mM methyl glyoxal for 2 h (c) 5 mM glucose for 30 min followed by addition of 5 mM MG for additional 1.5 h and (d) 5 mM methyl glyoxal for 2 h (d) wo/substrate for 2 h at 37°C. Figure S4. Detection of d- and l-lactate formation in erythrocytes at each time point during 2 h incubation using Yb3 (2.2 mM) by 1H NMR spectra of the supernatant of erythrocytes (40 % hematocrit) in phosphate buffered saline, pH 7.4 (a) 5 mM glucose for 2 h (b) 5 mM glucose for 30 min followed by addition of 5 mM MG for additional 1.5 h and (C) 5 mM methyl glyoxal for 2 h (d) wo/substrate for 2 h at 37°C. The data was normalized with no substrate incubation data as background correction. Figure S5. CEST spectra of the Yb3 complex (5mM) in RBC cultured media. Presaturation pulse of 5s with B1 of 15uT was applied at 298K using 9.4 NMR spectrometer: RBCs (40% hematocrit) was incubated in Phosphate-buffered saline (pH ~7.0, 2 h at 37 °C) with 5mM glucose; Glc 5mM, 5mM glucose 30min incubated then add 5mM methyl glyoxal; Glc + MG, and 5mM methyl glyoxal; MG 5mM. The CEST peaks are assignable at 168ppm to d-lactate and at 157ppm are l- lactate. Figure S6. 1H NMR (400MHz) spectra of media collected from (a) sgGlo1 pLHCX cells (re-expressed GLO1) (b) sgGlo1 (GLO1 deleted cells) and (c) parental 3353T3 cells. Each cell line was incubated with 5mM glucose in DMEM basal media for 4 h, 37 °C, pH 7. Subsequently, a 0.5 mL volume of media was collected and mixed with 10 mM Yb3 prior [file 40170_2021_267_MOESM1_ESM.docx]

Detection of glucose-derived D- and L-lactate in erythrocytes and cancer cells by use of a chiral NMR shift reagent

Eul Hyun Suh^1^, Carlos F. G. C. Geraldes^2,3^, Sara Chirayil^1^, Brandon Faubert^5^, Raul Ayala^4^, Ralph J. DeBerardinis^5,6,7^, and A. Dean Sherry^1,8,9**^

Affiliations:

1. Advanced Imaging Research Center, University of Texas Southwestern Medical Center, Dallas, TX, USA
2. Department of Life Sciences and Coimbra Chemistry Center, Faculty of Science and Technology, University of Coimbra, 3000-393 Coimbra, Portugal
3. CIBIT- Coimbra Institute for Biomedical Imaging and Translational Research, University of Coimbra, 3000-548 Coimbra, Portugal
4. School of Health Professions at Yvonne A. Ewell Townview Center, Dallas, TX, USA
5. Children’s Medical Center Research Institute, University of Texas Southwestern Medical Center, Dallas, TX, USA
6. Howard Hughes Medical Institute, University of Texas Southwestern Medical Center, Dallas, TX, USA
7. Department of Pediatrics and Eugene McDermott Center for Human Growth and Development, University of Texas Southwestern Medical Center, Dallas, TX, USA.
8. Department of Chemistry and Biochemistry, University of Texas at Dallas, Richardson, TX, USA
9. Department of Radiology, University of Texas Southwestern Medical Center, Dallas, TX, USA

**Binding affinity of Yb_3_ with D- or L-lactate**

The binding affinities of Yb_3_ toward D- or L- lactate were determined by ^1^H NMR. This experiment consists of measuring the ^1^H NMR intensity of Yb_3_ bound lactate CH_3_ resonances by increasing concentrations of the lactate at a fixed concentration of Yb_3_ complex (2.1mM). Increasing D- or L-lactate concentrations, from 0.4mM to 7mM, were added to 2.1 mM of Yb_3_ at pH 7.0. The areas of Yb_3_ bound CH_3_ resonances of D-lactate (^1^H δ = 21.0 ppm) and L-lactate (^1^H δ = 30.4 ppm) were measured and plotted against the Yb_3_ concentration. The respective association constants (*K*_A_) were calculated by fitting the data was fitted to equation S1 as follows:

${CH}_{3 Area}=C_{{Yb}_{3}}{\{\frac{1}{2}(I}_{0}) \times n\cdot C_{{Yb}_{3}}+C_{Lac}+{K_{A}}^{-1}\sqrt{{(n\cdot C_{{Yb}_{3}}+C_{Lac}+{K_{A}}^{-1})}^{2}-4n\cdot C_{{Yb}_{3}}\}}$

(Equation. S1)

Where $I_{0}$is the ^1^H NMR intensity for the lactate bound state, $C_{{Yb}_{3}}$ and $C_{Lac}$ are the concentrations of Yb_3_ and the D- or L-lactate, respectively. The number of binding sites ($n$ ) on the complex was assumed as 1.

Figure S1. Titration curves for D- and L-lactate

Figure S2. Full ^1^H NMR spectrum of D-lactate (15 mM) and L-lactate (15 mM) addition to Yb_3_ (6.3 mM) showing the presence of excess free lactate CH_3_ and CH resonances in their normal diamagnetic positions (inserted).


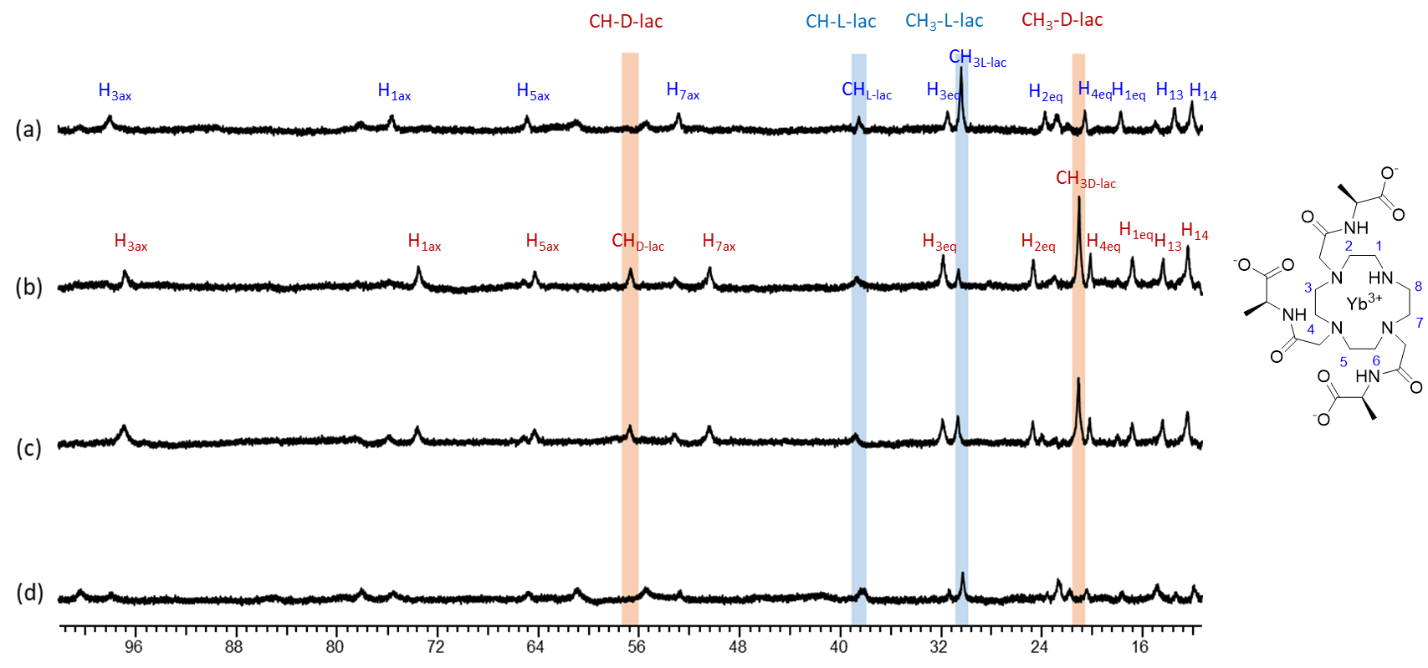


Figure S3. Detection of D- and L-lactate formation in erythrocytes using Yb_3_ (2.2 mM) by ^1^H NMR spectra of the supernatant of erythrocytes (40 % hematocrit) in phosphate buffered saline, pH 7.4 after incubation with (a) 5 mM glucose for 2 h (b) 5 mM methyl glyoxal for 2 h (c) 5 mM glucose for 30 min followed by addition of 5 mM MG for additional 1.5 h and (d) 5 mM methyl glyoxal for 2 h (d) wo/substrate for 2 h at 37°C.

|  | **L-lactate·Yb_3_** | **D-lactate·Yb_3_** |
| --- | --- | --- |
| H_3ax_ | 98.1 | 96.9 |
| H_1ax_ | 75.7 | 73.5 |
| H_5ax_ | 65.0 | 64.3 |
| H_7ax_ | 52.8 | 50.7 |
| H_3eq_ | 31.5 | 31.8 |
| H_2eq_ | 23.8 | 24.7 |
| H_4eq_ | 20.6 | 20.1 |
| H_1eq_ | 17.8 | 16.8 |
| H_14_ | 13.5 | 14.4 |
| H_13_ | 12.1 | 12.4 |
| CH_lact_ | 38.6 | 56.7 |
| CH_3lact_ | 30.4 | 21.0 |

Table. ^1^H NMR shift (ppm) of L-lactate·Yb_3_ and D-lactate·Yb_3_ of RBCs supernatant sample

Figure S4. Detection of D- and L-lactate formation in erythrocytes at each time point during 2 h incubation using Yb_3_ (2.2 mM) by ^1^H NMR spectra of the supernatant of erythrocytes (40 % hematocrit) in phosphate buffered saline, pH 7.4 (a) 5 mM glucose for 2 h (b) 5 mM glucose for 30 min followed by addition of 5 mM MG for additional 1.5 h and (C) 5 mM methyl glyoxal for 2 h (d) wo/substrate for 2 h at 37°C. The data was normalized with no substrate incubation data as background correction.


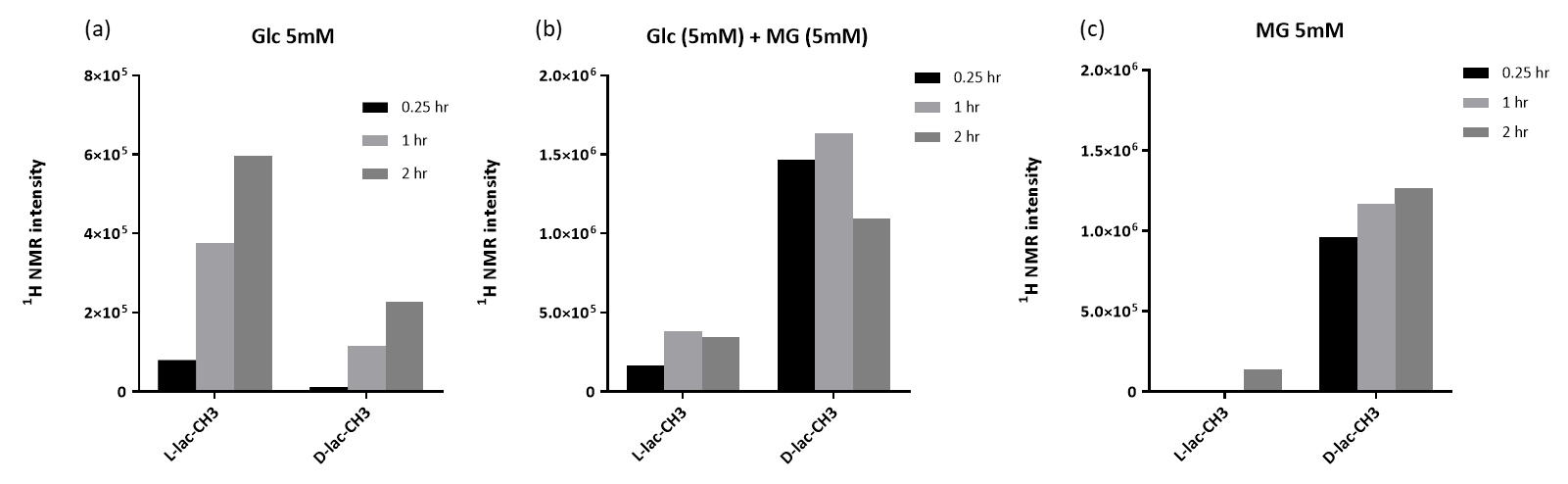

Figure S5. CEST spectra of the Yb_3_ complex (5mM) in RBC cultured media. Presaturation pulse of 5s with B_1_ of 15uT was applied at 298K using 9.4 NMR spectrometer: RBCs (40% hematocrit) was incubated in Phosphate-buffered saline (pH ~7.0, 2 h at 37 °C) with 5mM glucose ; Glc 5mM, 5mM glucose 30min incubated then add 5mM methyl glyoxal ; Glc + MG, and 5mM methyl glyoxal; MG 5mM. The CEST peaks are assignable at 168ppm to D-lactate and at 157ppm are L- lactate.

Figure S6. ^1^H NMR (400MHz) spectra of media collected from (a) sg*Glo1* pLHCX cells (re-expressed GLO1) (b) sg*Glo1* (GLO1 deleted cells) and (c) parental 3353T3 cells. Each cell line was incubated with 5mM glucose in DMEM basal media for 4 h, 37 °C, pH 7. Subsequently, a 0.5 mL volume of media was collected and mixed with 10 mM Yb_3_ prior to collection of the NMR spectra. The highlighted methyl resonances are assigned to L-(blue) and D-(red) lactate.


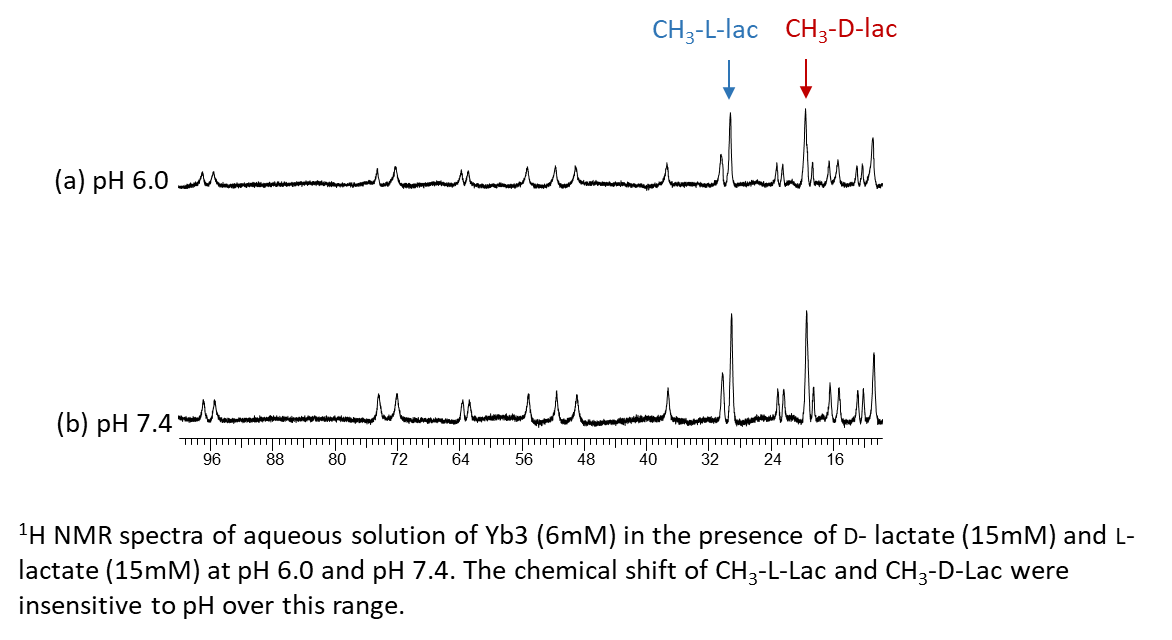


Figure S7. 1H NMR spectra of an aqueous solution of Yb_3_ (6 mM) in the presence of D-lactate (15 mM) and L-lactate (15 mM) at pH 6.0 and 7.4. The chemical shifts of CH_3_-L-lac and CH_3_-D-lac were insensitive to pH over this range. The signals of excess unbound D- and L-lactate appeared in their normal diamagnetic positions (not shown here).
